# Prophylactic routine posterior pericardiotomy: Should we perform it in every patient?

**DOI:** 10.1016/j.xjtc.2022.03.012

**Published:** 2022-04-15

**Authors:** Vasily I. Kaleda, Stepan S. Babeshko, Sergey Yu Boldyrev, Sergei A. Belash, Kirill O. Barbuhatti

**Affiliations:** a Department of Cardiac Surgery, Central Clinical Hospital, Moscow, Russia; b Department of Adult Cardiac Surgery, Ochapowski Regional Hospital #1, Krasnodar, Russia; c Division of Cardiology and Cardiac Surgery, Kuban State Medical University, Krasnodar, Russia

**Figure:**
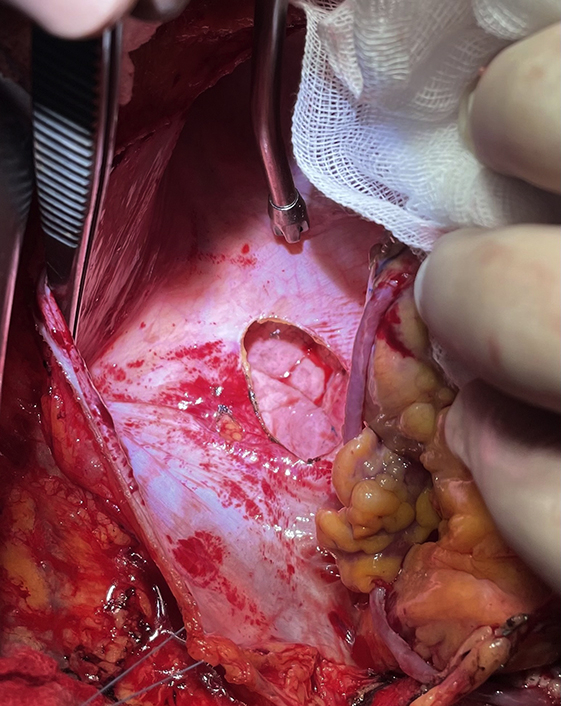
Posterior pericardiotomy prevents complications after cardiac surgery.

**Central Message**

Posterior pericardiotomy is a safe and simple procedure that may prevent not only postoperative atrial fibrillation, but also pericardial effusion and tamponade.

See Commentary on page 117.

During the American Heart Association Scientific Sessions 2021, Dr Mario Gaudino presented the results of the PALACS (Posterior Left pericardiotomy for the prevention of postoperative Atrial fibrillation after Cardiac Surgery) trial, which were simultaneously published in *The Lancet*.^1^ This trial attracted attention to posterior pericardiotomy (PP)—an intervention first applied in cardiac surgery a quarter-century ago,^2^ whose mechanism of action is linked to the elimination of pericardial effusion—a known trigger of atrial fibrillation.^3^ So, the question is raised: should this prophylactic intervention be applied in cardiac surgery routinely? To answer this question, we would like to briefly review the results of the PALACS trial and some earlier studies and our trial.

The authors of the PALACS trial conducted a perfectly designed and powered randomized controlled trial that has shown a beneficial effect of PP on preventing postoperative atrial fibrillation (POAF) in cardiac surgery. This trial included adult patients undergoing primary, elective coronary artery bypass grafting, aortic valve or ascending aortic procedures, or a combination of these, who had no previous history of arrhythmia. Patients undergoing mitral or tricuspid valve surgery were excluded from the study because, the authors stated, they had different pathophysiology and risk of POAF. However, whether patients undergoing isolated interventions on the coronary arteries have the same pathophysiology of POAF as do patients undergoing aortic valve or ascending aortic procedures is unknown. From several meta-analyses, we already know that PP is effective in patients undergoing isolated coronary artery bypass grafting.^4^^,^^5^ Now, from a prespecified subgroup analysis of the PALACS trial, we have learned that PP is also effective in aortic valve and ascending aortic procedures.

We also tried to answer whether PP is effective in patients undergoing aortic valve procedures. However, we received a completely different result that may add some information on the pathophysiology of POAF. Our randomized controlled trial included patients aged 18-69 years undergoing primary isolated aortic valve replacement.^6^ Besides preoperative atrial fibrillation, there was a long list of exclusion criteria, including the majority of known risk factors of atrial fibrillation and conditions that hinder PP performance: a history of thyroid dysfunction, amiodarone intake, pericardial effusion, severe chronic obstructive pulmonary disease, left ventricular ejection fraction <30%, left atrial systolic diameter >50 mm, active infective endocarditis, pericardial and/or pleural adhesions, and minimally invasive approach. Assuming a rate of POAF of 35% (a close number was used in an initial PALACS protocol^7^), we estimated that a sample size of 90 participants would have provided 80% power and 5% confidence level to detect a reduction of the primary outcome down to 11% (based on our systematic review^8^) in the PP group compared with the control group. In 2013—2015 we recruited 100 patients. The results of the study were surprising: the overall incidence of POAF was much lower than expected, and there was no difference between the groups in terms of both atrial fibrillation and pericardial effusion (Table 1).

**Table 1:** Patients and outcomes (full data available in Kaleda and colleagues^6^)

	Posterior pericardiotomy (n = 49)	Control (n = 51)	*Р* value
Age, y	56.6 ± 9.9	55.4 ± 10.5	.564
Sex (male)	28 (57%)	33 (65%)	.438
Cardiopulmonary bypass time, min	64 ± 16	64 ± 20	.664
Crossclamp time, min	45 ± 13	46 ± 12	.844
Mechanical prosthesis	35 (70%)	40 (80%)	.419
Chest tubes removal, POD	4.4 ± 1.4	3.3 ± 0.6	<.0001
Postoperative AF	8 (16%)	7 (14%)	.716
Onset of AF, POD	3.6 ± 2.5	3.0 ± 2.6	.551
AF at discharge	1 (2%)	1 (2%)	.977
Pericardial separation ≥5 mm	5 (10%)	6 (12%)	.803

Data presented as mean ± standard deviation and n (%). *POD*, Postoperative day; *AF*, atrial fibrillation.

We have 2 explanations for our results. First, we excluded patients with any known risk of atrial fibrillation, so we obtained a low incidence of this event in both groups, whereas, probably, PP works only in high-risk patients. Second, the results could be affected by our chest drainage strategy. While there is a clear trend to remove chest tubes as soon as possible (usually on the next day after surgery), we used a very conservative volume threshold for tube removal of 100 mL/day. This strategy led to really long chest tube duration—on the average of 4.4 days in PP group and 3.3 days in the control group (*P* < .0001). Consequently, in most patients the tubes (including the pericardial tube in the control group) were still in place at the time of greatest POAF risk. Early chest tube removal has been associated with an increased risk of pericardial effusion.^9^ Several years ago, an assumption was made that additional posterior pericardial tube may be as good as PP,^10^ and our results support this assumption. In fact, the effect of pericardial drainage using an additional tube placed posteriorly in the pericardium has been investigated. In a prospective randomized controlled trial by Eryilmaz and colleagues,^11^ a conventional 1-tube strategy was compared with a 2-tube strategy in patients undergoing ascending aortic surgery. The study showed that a thin drain placed retrocardially is effective in the prevention of posterior pericardial effusion. However, a dedicated prospective randomized controlled study by Sen and colleagues^12^ demonstrated the superiority of the right pericardial window (a technique that seems to be an analog of PP) over posterior pericardial tube in terms of reducing pericardial effusion. The superiority of PP over the posterior pericardial tube is easily explained by its longer functioning.

So, these 2 explanations of why PP did not work in our study may extend our understanding of the pathophysiology of POAF, but have no practical implications, because (1) nowadays most patients, such as those included in our study, are usually operated on via a minimally invasive approach, which hinders PP performance; so, recommendation not to perform PP in these patients would be meaningless, and (2) we cannot recommend prolonged chest drainage strategy because it hinders early postoperative physical activity and fast track recovery.

Interestingly, PP has been included in 2 clinical guidelines on prevention and management of POAF: by the American College of Chest Physicians (2005; *strength of recommendation, B; evidence grade, fair; net benefit, intermediate*)^13^ and the European Association for Cardio-Thoracic Surgery (2006; *grade B recommendation based on an individual level 1b study*).^14^ However, during the past 15 years, none of these documents has been updated.

To conclude, despite the findings of our trial, we believe that we should perform PP in every patient done through a full sternotomy and that the PALACS trial is an essential cornerstone on the way to a wider adoption of this technique in cardiac surgery. However, further research is needed to investigate the long-term effect of PP, as well as its effect in patients undergoing mitral and tricuspid valve procedures.

### Conflict of Interest Statement

The authors reported no conflicts of interest.

The *Journal* policy requires editors and reviewers to disclose conflicts of interest and to decline handling or reviewing manuscripts for which they may have a conflict of interest. The editors and reviewers of this article have no conflicts of interest.
